# Parthenolide and arsenic trioxide co-trigger autophagy-accompanied apoptosis in hepatocellular carcinoma cells

**DOI:** 10.3389/fonc.2022.988528

**Published:** 2022-10-24

**Authors:** Juan Yi, Xia Gong, Xiao-Yang Yin, Li Wang, Jin-Xia Hou, Jing Chen, Bei Xie, Gang Chen, Li-Na Wang, Xiao-Yuan Wang, Da-Chun Wang, Hu-Lai Wei

**Affiliations:** ^1^ School of Basic Medical Sciences, Lanzhou University, Lanzhou, Gansu, China; ^2^ Geriatrics Department, The First Hospital of Lanzhou University, Lanzhou, Gansu, China; ^3^ Biochemistry Department, LanZhou Ke Bao Biotechnology Co., Ltd., Lanzhou, Gansu, China; ^4^ Key Laboratory of Preclinical Study for New Drugs of Gansu Province, Lanzhou, Gansu, China

**Keywords:** arsenic trioxide, parthenolide, apoptosis, autophagy, hepatocellular carcinoma

## Abstract

Although arsenic trioxide (ATO) shows a strong anti-tumor effect in the treatment of acute promyelocytic leukemia, it does not benefit patients with hepatocellular carcinoma (HCC). Thus, combination therapy is proposed to enhance the efficacy of ATO. Parthenolide (PTL), a natural compound, selectively eradicates cancer cells and cancer stem cells with no toxicity to normal cells. In this study, we chose PTL and ATO in combination and found that nontoxic dosage of PTL and ATO co-treatment can synergistically inhibit the *in vitro* and *in vivo* proliferation activity of HCC cells through suppressing stemness and self-renewal ability and inducing mitochondria-dependent apoptosis. More importantly, USP7-HUWE1-p53 pathway is involved in PTL enhancing ATO-induced apoptosis of HCC cell lines. Meanwhile, accompanied by induction of apoptosis, PTL and ATO evoke autophagic activity *via* inhibiting PI3K/Akt/mTOR pathway, and consciously controlling autophagy can improve the anti-HCC efficacy of a combination of PTL and ATO. In short, our conclusion represents a novel promising approach to the treatment of HCC.

## Introduction

In the 1970s, the therapeutic potential of arsenic trioxide (As_2_O_3_, ATO) against acute promyelocytic leukemia (APL) was first recognized in China (1). Subsequently, the enormous clinical studies conducted in China and US confirmed that a low dose of ATO has shown strong therapeutic efficacy with little toxicity when used in the treatment of refractory and relapsed APL (2, 3). Thus, US Food and Drug Administration (FDA) approved ATO as a chemotherapeutic drug to treat APL in 2000 (4), which prompted researchers to examine the availability of ATO to various solid tumors (5–7). Many reports have shown that ATO exerts the antitumor effect in cell lines from solid tumors through multiple mechanisms, including inducing apoptosis (8–10), dampening proliferation (11–13), triggering partial differentiation (14, 15), directly damaging DNA (16, 17), suppressing tumor angiogenesis (18, 19) and metastasis (20, 21) and so on. However, our study and other studies proved that the effective dose of ATO used in solid tumors is much higher than that in APL (22–24). Furthermore, clinical investigations have revealed that ATO can be removed quickly in blood circulation and fail to accumulate effectively in solid tumors, leading to no response to the treatment with a nontoxic dose of ATO in patients with solid tumors (25). However, it is encouraging that patients with solid tumors, such as breast cancer, colorectal cancer, head-neck cancer and gliomas, benefited from ATO and other drug combination therapy in some completed clinical trials (26).

Human hepatocellular carcinoma (HCC) remains a severe global health threat by reason of high mortality and recurrence. Although surgery is an option as first-line therapy for patients with HCC, systemic chemotherapy is still critical to keep the patients from relapsing after surgery. Given the powerful effects of ATO against APL, ATO was introduced to treat HCC cell lines in laboratory and found to be able to induce apoptosis and cell growth. But in fact, a phase II clinical trial verified that high dose ATO administered was not good for HCC patients and adversely caused grade 4 hematological toxicities (27). Hence, the establishment of therapeutic combination is required to improve the efficacy of nontoxic dose of ATO in HCC.

Parthenolide (PTL), a natural herbal compound from feverfew (28), has a significant anti-tumor effect in all types of cancers, but hardly affects normal cells (29, 30). More importantly, PTL is the first agent that specifically kills tumor stem cells (29, 31). As for the anti-tumor mechanisms of PTL, existing evidences have shown that it can directly suppress NF-κB pathway and deubiquitinase USP7 activated abnormally in cancer cells and tumor stem cells (32, 33). Of note, our previous study and other group study showed that PTL can enhance the sensitivity of tumor cells to drugs (34–36), indicating PTL may be an ideal candidate to sensitize solid tumor cells to ATO. As expected, Wang et al. (22) elucidated that the combination treatment with PTL and ATO significantly reduced growth of pancreatic cancer compared with those treated with either PTL or ATO alone. Thus, it is worth to explore whether PTL improves the efficacy of ATO in HCC.

In clinical chemotherapy, drug resistance is the most common event and causes recurrence and failure of treatment in HCC. Autophagy, a complex process of transferring cellular substances to lysosomes for degradation in normal or stressed conditions, contributes to the occurrence of drug resistance. Mounting studies have shown that almost all of chemotherapeutics cause changes in the activity of autophagy, and inhibiting autophagy can improve the efficacy of drugs (37–40). However, the existing reports mainly focus on the single drug-induced autophagy, and the roles of autophagy in combination of drugs is unclear.

Herein, we found that PTL combined with ATO synergistically inhibits the *in vitro* and *in vivo* proliferation activity of HCC cells through suppressing stemness and self-renewal ability and inducing mitochondria-dependent apoptosis, in which USP7-HUWE1-p53 pathway involved. Accompanied by induction of apoptosis, PTL and ATO evoke autophagic activity by inhibiting PI3K/Akt/mTOR pathway, and consciously controlling of autophagy can accelerate the anti-HCC efficacy of combination of PTL and ATO. In short, our conclusion represents a novel promising approach in the treatment of HCC.

## Materials and methods

### Drugs and antibodies

Drugs used in this study: Chloroquine (CQ; Sigma-Aldrich, C6628), Arsenic trioxide (ATO; Beijing Chemical, GB673-77) and Parthenolide (BIOMOL, P8522f). Antibodies specific against Caspase3 (9662), cleaved Caspase3 (9661), Pho-p53 (9284), Cdc25 (3652), p21 (2947), CyclinB (12231), Pho-S6K (323), CyclinE1 (20808S), ATG5 (12994), PI3K (4263), Akt (9272), Pho-Akt (13038), Pho-mTOR (5536), Pho-4EBP1 (2855) and BCL-2 (15071) were purchased from Cell Signaling Technologies. Antibodies specific against BAX (sc493), p53 (sc126), catalase (sc50508) and SOD1 (sc11407) were purchased from Santa Cruz. Antibodies specific against HUWE1 (ab70161) and USP7 (ab4080) were purchased from Abcam. Other antibodies were used in this study: CD133 (Immunway, YT5192), GFP (Thermo fisher scientific, MA5-15256), LC3B (Sigma, L7543), mTOR (Genetex, 41731) and GAPDH (Proteintech, 60004-1-Ig). Secondary antibodies used in this study: Peroxidase-conjugated affinity pure goat antimouse IgG, light chain specific (Jackson Immuno Research, 115–035-174), peroxidase-conjugated IgG fraction monoclonal mouse antirabbit, light chain specific (Jackson Immuno Research, 211–032-171).

### Cell culture

HEK293T, HepG2, MHCC 97H and Huh7 cells were cultured in Dulbecco’s modified Eagle’s medium (DMEM; HyClone, SH30022.01)) supplemented with 10% fetal bovine serum (FBS; YEASEN, 40130ES76) and 1% Penicillin/Streptomycin, LO2 cells and H22 cell were cultured in Roswell Park Memorial Institute 1640 (RPMI1640; HyClone, SH30027.01) supplemented with 10% FBS and 1% Penicillin/Streptomycin. All cells were incubated in a humidified 5% CO_2_ incubator.

#### Cell viability assay

5×10^3^ cells were seeded in 96-well plates. After treatment with ATO and/or PTL for the indicated times, 10 μL MTT solution was added to each well. Plates were incubated for an additional 4 h at 37°C,100 μL % SDS was added to each well to solubilize formazan crystals. Each sample point was assayed with 4 replica points. Optical density was measured at 570nm using a microplate reader (Powerwave X, Bio-Tek) to calculate inhibition rate for cell proliferation and 50% inhibitory concentration (IC50).

#### Cell apoptosis

After the designated treatment, cells were washed and labeled with Annexin V/PI kit (eBioscience, 800-8005-72) for 15 min at room temperature in the dark, according to the manufacturer’s instructions. The images were captured using fluorescence microscopy (OLYMPUS, IX81), and the percentage of apoptosis was measured by Flow cytometry (Beckman-Coulter Epics XL).

#### Cell cycle analysis

Cells were treated with drugs at the indicated concentrations for 24 h, and then collected and fixed with 75% ethanol at -20°Covernight. Cells were washed twice with cold PBS and resuspended in propidium iodide (PI; Solaibio, C0080)) staining buffer containing RNAse. Following incubation for 30 min at room temperature in the dark, cells were analyzed by Flow cytometry.

#### Mitochondrial membrane potential

Cells were seeded into a 96-well plate and treated for 24 h with drugs at indicated concentrations. After treatment, cells were incubated at 37°C with TMRE (100 nM) (Thermo Fisher Scientific, I 34361) in growth media for 30 min in a CO_2_ incubator. After incubation, cells were analyzed using fluorescence microscopy to determine the fluorescence intensity. Measurement of TMRE integrated intensity was done using Image J software.

#### Mitochondrial reactive oxygen species measurement

Cells were seeds into 96-plate and treated with ATO, PTL and ATO plus PTL for 24 h, followed by incubation with MitoSOX™ red mitochondrial superoxide indicator (Invitrogen, M36008) for 15min at 37°C. Cellular fluorescence intensity was detected using fluorescence microscope. The fluorescence intensity quantified by ImageJ software.

#### Colony formation assay

HpeG2 cells were seeded in six-well plates at low density (2,000 cells per well). In next day, ATO, PTL and ATO plus PTL was added directly into each well at indicated concentration. After 12 days, the plates were washed with PBS and stained with coomassie brilliant blue. The number of colons as counted and the morphology of single clone was observed under the microscope (>50 clones validated).

#### Plasmids

EYFP-USP7 constructs was generated using PCR amplification and subcloned into pEYFP-C1 vectors using ClonExpress II one step cloning Kit (Vazyme, C112-01). pEYFP-C1was obtained from Clontech. LentiCRISPRv2 puro (Addgene, 98290) was a gift from Brett Stringer (41). PMD2.G (Addgene, 12259) and psPAX2 (Addgene, 12260) were gifts from Didier Trono.

#### Establishment of ATG5 KO cell lines using CRIPSR/Cas9 system

ATG5 guide RNAs were designed with the online CRISPR design tool. The gRNA sequences (sg-ATG5-1: 5′-caccggatggacagttgcacacact-3′, sg-ATG5-2: 5′-aaacagtgtgtgcaactgtccatcc-3′) were purchased from Sangon Biotech. These sgRNAs were cloned into LentiCRISPRv2 according to the protocol provided by the Zhang Lab (42). These two plasmids containing the guide RNA of interest and packaging plasmids (PMD2.G and psPAX2) were transfected into HEK293T cells. After 96 h, virus supernatant was harvested, and then concentrated using Lentivirus concentration solution (YEASEN, 41101ES50). HepG2 cells were infected with concentrated virus supernatant for 48 h. Followed by puromycin (400 ng/ml; Merck, P9620) selection for 2 weeks. The ATG5 knockout effect in puromycin-resistant cells was then verified by immunoblotting.

#### Western blotting

After designated treatment, Laemmli buffer (62.5 mM Tris-HCl, pH 6.8, 20% glycerol, 2% SDS, 2 mM dithiothreitol (DTT), phosphatase inhibitor and proteinase inhibitor cocktail [Thermo Fisher Scientific, 78446]) was used to lyse the harvested cells. An equal amount of protein was separated on SDS-PAGE gels and then transferred onto PVDF membranes (Bio-Rad Laboratories, 1620177). After blocking with 5% nonfat milk in TBST, membranes were incubated with the indicated primary antibodies and secondary antibodies, and then visualized using Super ECL Detection Reagent (YESEN, RPN2232) with CLINX Hemiscope (QinXiang, China).

#### Animal studies

Kunming mice and BALB/c mice (8 weeks, 200-250g) were approved by Laboratory Animal Science and Technology Work Management Committee, School of Basic Medicine, Lanzhou University, which is fully accredited by the Institutional Animal Care and Use Committee. For xenograft experiments, H22 cell were injected into the abdominal cavity of 8-week-old Kunming mice. One week later, we collected ascites and counted 5×10^5^ cells in 200 μL PBS for subcutaneous injection into left flanks to establish tumors. Treatment was started when tumor volumes reached a minimal size (notable by sight, approximately a week after injection). Mice were treated with drugs by intraperitoneal injection at the indicated concentrations for 14 times in 28 days, and mice showed no toxicity to all drugs after injection. After 28 days, the mice were sacrificed and tumors were harvested.

#### Statistical analysis

All numerical data are expressed as the mean ± SD from at least three independent experiments. Statistical significance of differences between groups was evaluated using Student’s t-test. Statistical analysis was performed using SPSS statistical software (version 21.0). *P*<0.05 was considered to be statistically significant.

## Results

### Further suppression of proliferation of HCC cells by combination of PTL and ATO

**Figure 1:**
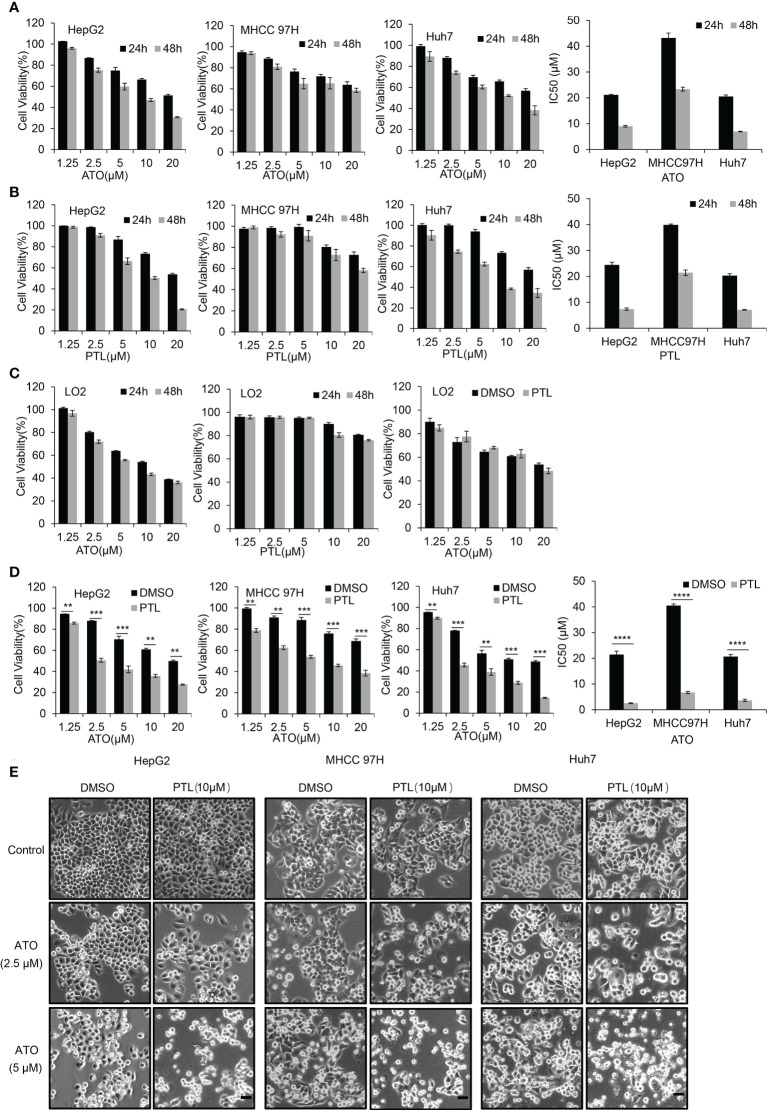
PTL enhances the effect of growth suppression of HCC cell lines mediated by ATO without influence on the normal hepatocyte LO2 cells. **(A, B)** MTT assay showing the viability of HepG2 cells, MHCC97H cells and Huh7 cells after exposure to increasing concentrations of ATO or PTL for 24 h and 48 h. The half-maximal inhibitory concentration (IC50) of ATO or PTL was calculated (*P* < 0.0001 vs. Control). **(C)** LO2 cells were treated different concentrations of ATO, PTL or ATO plus PTL for 24 h or 48 h, and the cell viability was measured by MTT assay. **(D)** After exposure to indicated concentrations of ATO, PTL and ATO plus PTL for 24 h, the cell viability was tested by MTT assay and IC50 was calculated (**P < 0.01; ***P < 0.001; ****P < 0.0001). **(E)** After exposure to indicated concentrations of ATO, PTL and ATO plus PTL for 24 h, the changes of cell morphology were observed under microscope. Scale bar: 20 μm.

Based on metastatic properties, three different HCC cell lines, HepG2 cells, MHCC 97H cells and Huh7 cells, were used to separately evaluate the effect of ATO or PTL on HCC cells growth. MTT assay showed that both drugs dramatically inhibited the proliferation of three cell lines in a dose and time dependent manner. The half inhibitory concentrations (IC50) of ATO are 21.2 μM and 9.0 μM (HepG2), 43.2 μM and 23.4 μM (MHCC 97H), 20.5 μM and 6.9 μM (Huh7) for 24 h and 48 h respectively (**Figure 1A**). The IC50 of PTL are 24.5 μM and 7.4 μM (HepG2), 39.9 μM and 21.4 μM (MHCC 97H), 20.3 μM and 7.1 μM (Huh7) for 24 h and 48 h (**Figure 1B**). According to our previous studies and other groups evidences, we found that HCC cell lines were less sensitive to ATO or PTL than leukemia cells (the value of IC50 is 3.4 uM for 24 h) (24). In a separate study, we assessed the cellular toxicity of ATO and PTL to human hepatocyte LO2 cells. The results indicated that ATO had appreciable toxicity at the doses treated HCC cell lines, whereas, we observed no obvious effect to LO2 cells at the doses that HCC cell lines demonstrated strong specific toxicity in response to PTL. In following experiments, the influences of PTL on ATO-mediated cytotoxicity was separately investigated in three HCC cell lines and LO2 cells using MTT assay. The data showed that the combination of ATO and PTL did not increase the ATO-induced toxicity to LO2 cells (**Figure 1C**). Conversely, HCC cell lines showed more remarkable suppression of cell growth in response to ATO/PTL combined group than each agent alone group. Consistent with this result, we observed combined treatment yielded a significantly higher reduction in cell numbers than those obtained with either drug alone using contrast microscope (**Figures 1D, E**). Taken together, these results suggested that PTL may decrease the effective dose of ATO required for treatment of hepatocarcinoma cells.

#### PTL and ATO co-induces mitochondria-dependent apoptosis in HCC cells

**Figure 2:**
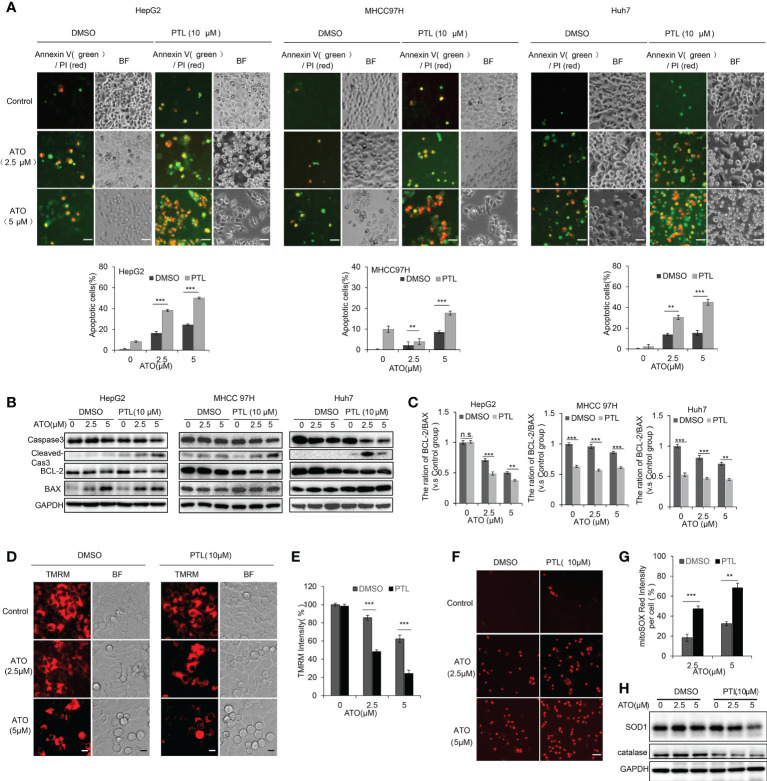
PTL promotes the cell apoptosis of HCC cell lines mediated by ATO *via* weakening the mitochondrial membrane potential and elevating ROS. Exposure to indicated concentrations of ATO, PTL and ATO plus PTL for 12 h or 24 h. **(A)** Apoptotic cells were analyzed by fluorescence microscope and Flow cytometry (**P < 0.01; ***P < 0.001). **(B)** The cells were harvested and the indicated proteins were analyzed using western blotting, the expression of proteins were quantified as in **(C)**; Columns, mean; bars, ± S.D. of 3 independent experiments (**P < 0.01; ***P < 0.001; n.s, no significance). **(D)** HepG2 cells were stained with TMRM and the images were captured using fluorescence microscope. Scale bar: 10 μm. **(E)** The relative intensity of TMRM in the samples was quantified (n = 200). Columns, mean; bars, ± S.D. of 3 independent experiments (***P < 0.001). **(F)** HepG2 cells were stained with mitoSOX and observed using fluorescence microscope. Scale bar: 50 μm. The relative intensity of mitoSOX in the samples was quantified (n = 200) as shown in **(G)**; Columns, mean; bars, ± S.D. of 3 independent experiments (**P < 0.01; ***P < 0.001). **(H)** HepG2 cells were harvested and analyzed using indicated antibodies.

To ascertain the consequence of combined treatment with ATO/PTL in HCC cell lines, we assessed the changes of cell apoptosis. Following a 24h-treatment with ATO and/or PTL, the apoptotic rate was examined using Annexin-V/PI double staining. Fluroscence image showed that apoptotic cells were significantly elevated in ATO plus PTL group in three HCC cell lines, and FCM analysis demonstrated that 16.37%, 24.53% and 8.27% apoptotic rate in 2.5 μM alone, 5 μM ATO alone and 10 μM PTL alone treatment group, whereas combined treatment resulted in a 2~3-fold increase in the apoptotic rate of the HepG2 cells. Consistent results were also observed in MHCC 97H and Huh7 cells (**Figure 2A**). Subsequently, we also observed the significant elevation of cleaved caspase-3 accompanied with a reduction in the level of the total caspase-3 after the combination treatment in HCC cell lines. Based on these evidences, we concluded that combined treatment of ATO and PTL is more effective for induction of apoptosis in HCC cell lines (**Figure 2B**). Additionally, compared with alone reagent treatment, co-treatment with ATO and PTL showed a more obvious decline in anti-apoptotic protein BCL-2 and a more dramatic enhancement in pro-apoptotic protein BAX (**Figures 2B, C**). In follow-up investigation about the mechanisms of ATO/PTL-induced apoptosis, we observed that PTL/ATO combined treatment led to an appreciable reduction of the mitochondrial membrane potential (ΔΨm), an early trigger of mitochondrial apoptotic pathway, using TMRM staining (**Figures 2D, E**). Next, we identified the intracellular ROS using mitoSOX, and the images showed an increase in the release of ROS after treatment with ATO and PTL together (**Figures 2F, G**). To confirm this increase, the expression of superoxide dismutase 1 (SOD1) and catalase were examined using western blotting. The results indicated that combined treatment caused a more visible decrease in these two protein levels than the reagent alone (**Figure 2H**).

### PTL promotes the blockage of cell cycle in G2/M phase endowed by ATO

**Figure 3:**
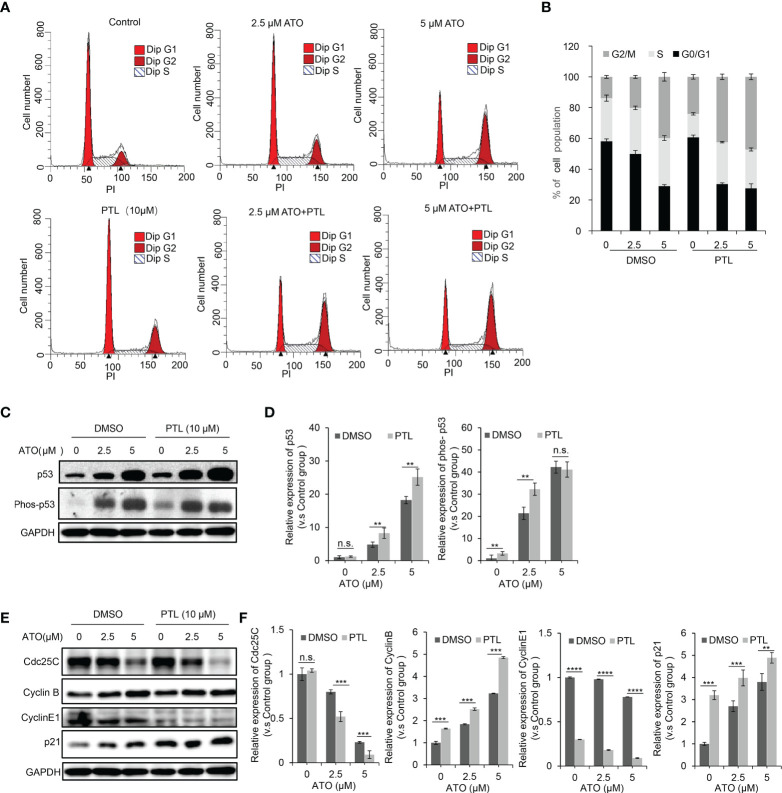
PTL worsened blockage of the cell cycle induced by ATO. Exposure to indicated concentrations of ATO, PTL and ATO plus PTL for 24 h. **(A)** The distribution of cell cycle in HepG2 was measured using Flow cytometry after PI staining. The percentage of G_0_G_1_, S and G_2_M were quantified as in **(B)**. **(C)** HepG2 cells were harvested and analyzed with indicated antibodies, and the expression of proteins were quantified as in **(D)**; Columns, mean; bars, ± S.D. of 3 independent experiments (**P < 0.01; n.s., no significance). **(E)** HepG2 cells were harvested and analyzed with indicated antibodies, and the expression of proteins were quantified as in **(F)**; Columns, mean; bars, ± S.D. of 3 independent experiments (**, *P* < 0.01; ***P < 0.001; ****P < 0.0001; n.s., no significance).

PTL or ATO are known to affect cell proliferation by arresting cell cycle progression (23, 43). To test whether combination of ATO and PTL more effectively blocked cell cycle than each reagent alone, we next performed the cell cycle analyses following treatment with ATO and/or PTL for 24h and observed a slight block at the G1 phase for PTL alone, a significant arrest at G2/M phase for ATO alone, and a much more block at G2/M for co-treatment with ATO/PTL (**Figures 3A, B**). p53 negatively regulates cell cycle progression in response to different cellular stresses, whereas phosphorylated p53 represents the activation of p53 (44). Thus, we examined the changes in expression of p53 and phosphorylated p53. The western blotting results showed that there was a dose-dependent increase in p53 and phosphorylated p53 in response to ATO alone, whereas combination with ATO and PTL showed approximate two folds improvement in the protein level of both compared with ATO alone (**Figures 3C, D**). In further study, the expression of proteins involved in the G1 and G2/M checkpoint was examined. After treatment with ATO and PTL alone, or co-treatment with ATO and PTL, the expression of Cdc25C, Cyclin B, Cyclin E, and p21 was measured by western blotting (**Figures 3E, F**). The results showed that the expression of Cdc25C were significantly reduced and p21 protein levels were enhanced in the combination group. Cyclin E, a checkpoint protein of G1 phase, was remarkably reduced after treatment with PTL alone, but had no obvious differences between PTL alone group and co-treatment group. However, the checkpoint protein of G2/M phase, Cyclin B, was enhanced after treatment with ATO alone or ATO and PTL. The probability that upstream Cdc25C was dramatically down-regulated, leading to the accumulation of Cyclin B. Taken together, these results demonstrated that combination with ATO and PTL is more effective to arrest cell cycle progression at G2/M phase.

### PTL and ATO synergistically suppress stemness and self-renewal ability of HepG2 cells

**Figure 4:**
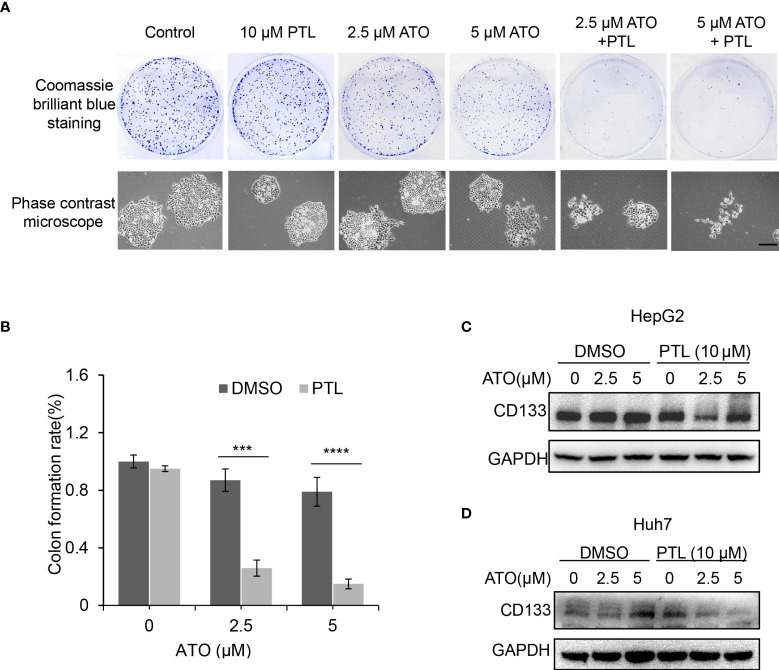
Suppression of stemness and self-renewal ability of HepG2 cells by combination of ATO with PTL. **(A)** HepG2 cells were treated with ATO, PTL and ATO plus PTL. After 12 days, cell clones were stained with coomassie brilliant blue. The number of colones was counted and the morphology of single clone was observed under the microscope (>50 clones validated). **(B)** Graphic quantitation of clone was obtained from at least three independent experiments. Columns, mean; bars, ± S.D. (***P < 0.001; ****P < 0.0001). **(C, D)** HepG2 cells and Huh7 cells were treated as **(A)**, and then subjected to western blotting analysis.

PTL is known as the first natural compound that specially eradicated cancer stem cells (30). And self-renewal is the ability by which stem cells divide to make more stem cells. In next experiment, clone formation assay was performed to test the changes in self-renewal ability to assess the anti-tumor of PTL/ATO combination *in vitro*. The CBB staining showed a significant reduction in the number of clone in ATO/PTL combined treatment group compared to ATO alone treatment group. Using microscope, we also observed the distinct shrinkage in the morphology of clone in combination group compared to each reagent alone group (**Figures 4A, B**). CD133 is one of the most well-characterized bio-markers used for the isolation and identification of cancer stem cells. In further study, we assessed the changes of CD133 protein level to clarify the effect of combined treatment on cancer stem cells. As we expected, the combination of two reagents apparently lowered the protein level of CD133 in contrast to each reagent (**Figures 4C, D**). Altogether, these data indicated that self-renewal ability is more effectively inhibited by ATO co-treated with PTL in HCC cell lines, suggesting that PTL may eliminate the cancer stem cells to exert the effect of ATO-mediated anti-tumor.

### USP7-HUWE1-p53 axis is involved in PTL enhancing ATO-induced apoptosis of HCC cell lines

**Figure 5:**
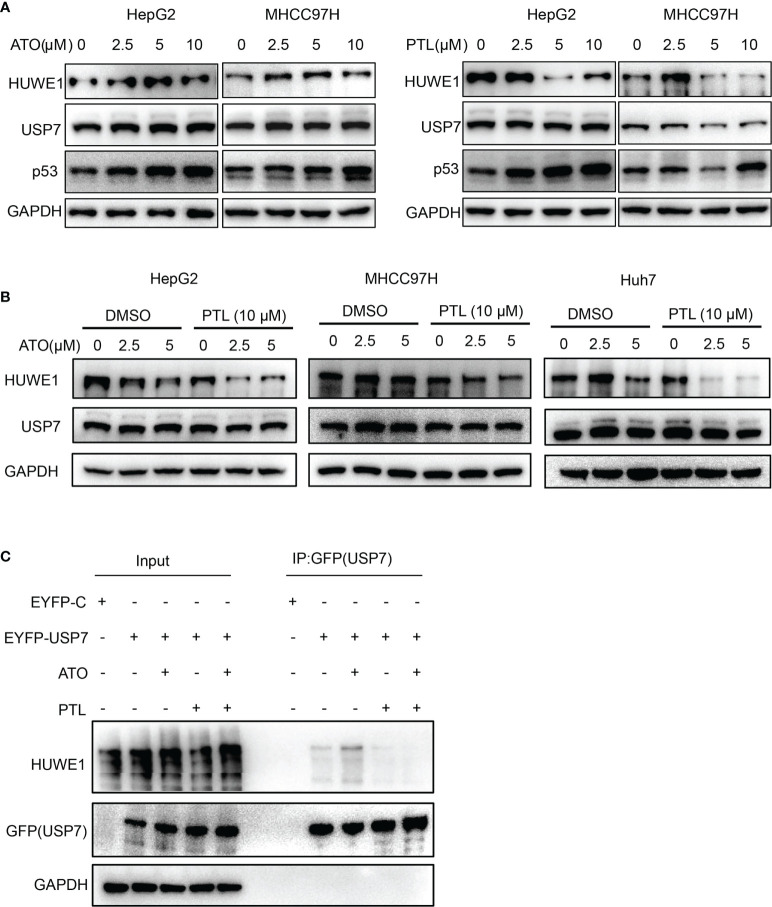
USP7-HUWE1 is related to PTL enhancing ATO-induced apoptosis of HCC cell lines. **(A)** HepG2 cells and MHCC 97H cells were treated with indicated concentrations of ATO or PTL for 24 h. The cells were collected and subjected to western blotting. **(B)** HepG2 cells, MHCC97H cells and Huh7 cells were treated with ATO, PTL and ATO plus PTL for 24 h, followed by analysis with western blotting. **(C)** Designated plasmids were transfected into HepG2 cells for 24 h, and then cells were treated with indicated concentrations of ATO or TL for 24 h. Harvested cells were lysed with IP lysis buffer and subjected to IP using GFP-agrose and western blotting analysis with the indicated antibodies.

The latest findings proved that PTL can directly interact with USP7 and inhibit its protein activity. HUWE1, an E3 ligase, is one of the substrates of USP7, and is responsible for the degradation of p53 (33, 45, 46). Therefore, we next explored whether USP7-HUWE1-p53 axis is involved in ATO/PTL -mediated apoptosis. The expression of USP7, HUWE1 and p53 was first detect in these cells treated with ATO or PTL alone at different concentrations using Western blot. The results showed that ATO had no apparent effect on expression of HUWE1 and only increased the protein level of p53, whereas, PTL exhibited a significantly decrease in protein level of HUWE1 going with enhancement of p53 (**Figure 5A**). These data gave us a hint that the inhibition of USP7 by PTL may improve the sensitivity of ATO. To confirm this hypothesis, we tested the changes of HUWE1 and p53 after treatment with ATO and PTL together. The results showed that compared with reagent alone, the combined use of ATO and PTL made the expression of HUWE1 decrease (**Figure 5B**), indicating ATO combination with PTL promotes the apoptosis of liver cancer cells through the USP7-HUWE1-p53 axis. At the same time, we evaluated the interaction between USP7 and HUWE1 after the combined action of drugs through the immunoprecipitation experiment (**Figure 5C**). The results showed that the interaction between USP7 and HUWE1 was significantly weakened after the co-treated with PTL compared with ATO alone. The above results prove that the effect of PTL on ATO-induced apoptosis of HCC cells may be achieved by regulating the USP7-HUWE1-p53 axis.

### PTL enhances ATO-triggered protective autophagy by PI3K/Akt/mTOR pathway in HCC cell lines

**Figure 6:**
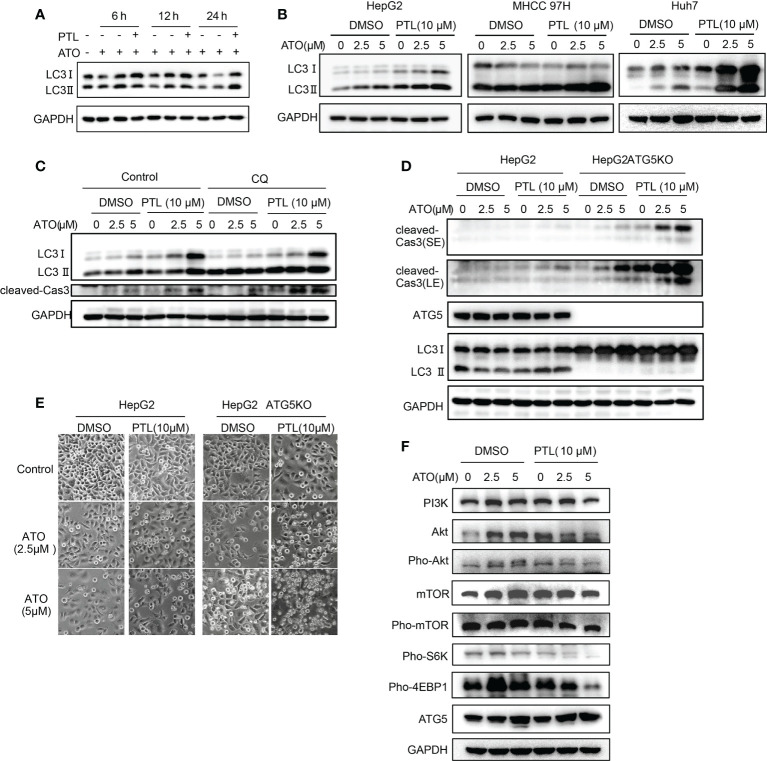
PTL enhances ATO-triggered protective autophagy by PI3K/Akt/mTOR pathway in HCC cell lines. **(A)** HepG2 cells were treated with ATO, PTL and ATO plus PTL for 6 h, 12 h and 24 h. Cells were harvested and subjected to western blotting analysis. **(B)** HepG2 cells, MHCC97H cells and Huh7 cells were treated with ATO, PTL and ATO plus PTL for 24 h. Cells were harvested and subjected to western blotting analysis. **(C)** HepG2 cells were treated with ATO, PTL and ATO plus PTL for 24 h with or without 50 μM CQ. Cells were harvested and subjected to western blotting analysis. **(D)** HepG2 cells and HepG2 ATG5 KO cells were treated with ATO, PTL and ATO plus PTL for 24 h. Cells were harvested and subjected to western blotting analysis. **(E)** HepG2 cells and HepG2 ATG5 KO cells were treated with ATO, PTL and ATO plus PTL for 24 h. The changes of cell morphology were observed under microscope. Scale bar: 20 μm. **(F)** HepG2 cells were treated with ATO, PTL and ATO plus PTL for 24 h. Cells were harvested and subjected to western blotting analysis.

To identify whether the combination of ATO/PTL has an impact on activity of autophagy, we first determined the best time point of activation of autophagy *via* examining the conversion of autophagy marker LC3. As shown by western blot, the best time point of autophagy activation is at 24 h after treatment with the combination ATO/PTL (**Figure 6A**). In follow-up investigation, we observed that the combination treatment had more higher conversion of LC3Ito LC3II compared with reagent alone in HCC cell lines (**Figure 6B**), indicating much stronger ability of autophagic induction in ATO/PTL combination group. LC3 immunostaining showed the similar consequence (**Figure S1A**). Next, we investigated the role of this increased autophagy in the process of drug combination. Cells were treated with ATO and PTL in absence or presence of CQ, and western blot results demonstrated that suppression of autophagy showed a significant increase in the level of cleaved caspase3, suggesting that activated autophagy have a protective effect on tumor cells as well as drug alone (**Figure 6C**). In addition, we further confirmed this conclusion in the ATG5- KO HepG2 cells (**Figure 6D**). Subsequently, we assessed the anti-tumor effect of drugs *in vitro* and observed that knock out ATG5 had a remarked promotion of cell death in whatever either drug group or combination of both drugs (**Figure 6E**). Finally, to elucidate the underlying mechanism of autophagy activation, we investigated the changes of some vital proteins in PI3K/Akt/mTOR signal pathway, an important pathway in the treatment of liver cancers and autophagy (47, 48). Our results showed that the combination of both drugs produced an abrogation in the expression levels of PI3K, AKT, mTOR, phosphorylated Akt and phosphorylated mTOR compared with either reagent. Furthermore, phosphorylation of S6K1 and 4EBP1, the downstream effectors in PI3K/AKT/mTOR signal pathway, also decreased in the combined group (**Figure 6F**). Taken together, these data indicated that the combination of ATO/PTL endows the HCC cell lines with much stronger autophagic activity than each reagent *via* inhibiting the PI3K/Akt/mTOR pathway, which may weaken the anti-tumor efficacy of the combination of PTL and ATO.

### PTL synergizes ATO to inhibit the *in vivo* growth of hepatoma H22 cells in mouse HCC model

**Figure 7:**
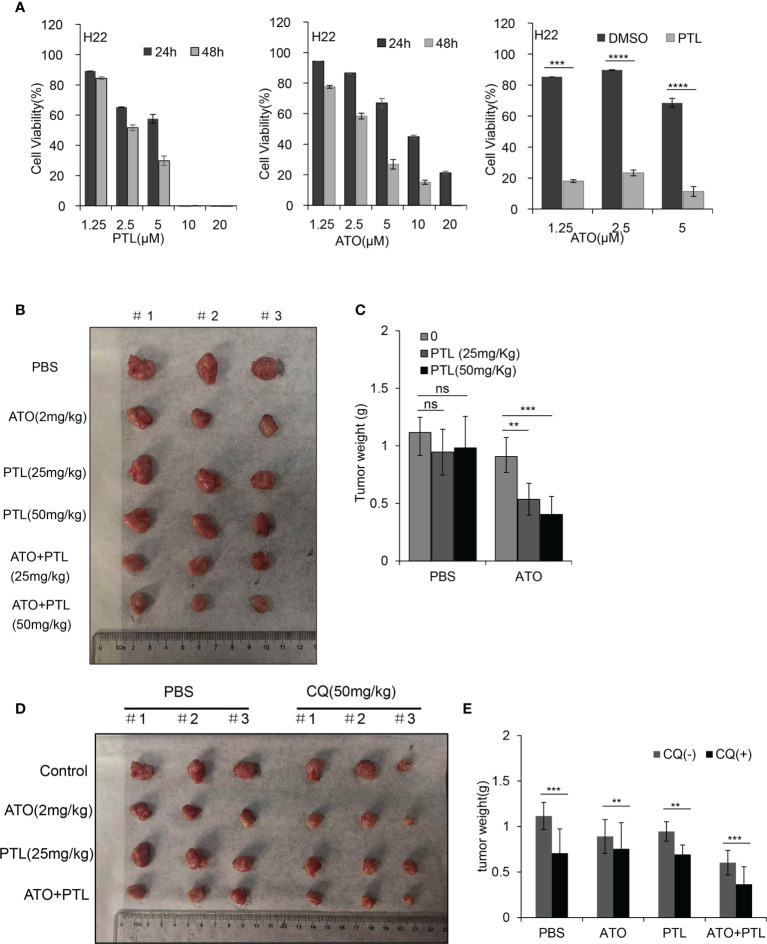
Effects of the combination of ATO with PTL on hepatoma growth. **(A)** MTT assay showing the viability of H22 cells after exposure to increasing concentrations of ATO or PTL for 24 h and 48 h (*P* < 0.0001 vs. Control). After exposure to indicated concentrations of ATO, PTL and ATO plus PTL for 24 h, the cell viability was tested by MTT assay. Columns, mean; bars, ± S.D. (***P < 0.001; ****P < 0.0001). **(B, D)** Photographs of harvested xenograft tumors prior to processing. **(C, E)** The weight of xenograft tumors in mice are shown. Columns, mean; bars, ± S.D.(n=3) (**P < 0.01; ***P < 0.001).

In the present study, we intended to evaluate the potential therapeutic efficacy of combination treatment with ATO, PTL and CQ in a xenograft Balb/c tumor model with subcutaneously implanted H22 cells, a mouse HCC cell line. Before establishing the animal model, we wondered whether PTL enhances the susceptibility of H22 cells to ATO as well as human HCC cell lines. As we expected, ATO or PTL significantly inhibited the proliferation of H22 cells, and the inhibition of cell growth by ATO was further enhanced after the addition of PTL (**Figure 7A**). Thus, H22 cells is suitable for the establishment of xenograft in this study. After the xenograft Balb/c tumor model was subjected to 28-day-treatment of ATO or PTL alone, the tumor growth was partially delayed compared with controls, whereas ATO combined with different concentrations of PTL strongly suppressed tumor growth (**Figures 7B, C**). Further, we observed that application of CQ significantly accelerated the decrease of the tumor growth caused by ATO and PTL (**Figures 7D, E**). Anyway, the observed data suggested that PTL synergistically accelerated the *in vivo* anti-HCC efficacy of ATO, and suppression of autophagic activity further promoted the therapeutic effects of PTL and ATO.

## Discussion

ATO, a Chinese traditional medicine, has shown substantial efficacy in treating patients with APL. More interestingly, our previous study revealed that multidrug resistant leukemia cells have much more sensitivity to ATO than the parallel sensitive cells, showing the unique feature of ATO distinguished from other chemotherapeutics (1, 24). In view of these, our attention is attached to the application of ATO in solid tumors. HCC is a malignant tumor with poor prognosis. The selection of chemotherapeutic agents is an important part of therapy to patients with HCC. In this study, ATO was introduced to assess the efficacy of anti-HCC. During research, however, we found IC50 of ATO in HCC is about ten times higher than that in leukemia cells, suggesting high dose of ATO required for HCC have the risk of toxicity and single-agent do not benefit patients with HCC. This urges us to consider whether treatment benefit emerges when ATO is combined with other agents. At this moment, we face a critical problem: How to choose the drugs combined with ATO? Ideally, this kind of drugs should have the features of nontoxicity and antitumor activity. PTL, a natural compound, has been reported to possess the powerful ability of killing cancer cells and cancer stem cells, and have no toxicity to normal cells. In published paper, we elaborated that PTL can increase the sensitivity of drug resistant leukemia cells to doxorubicin *via* impeding the expression of multi-drug resistance proteins (34). Therefore, we selected the combination of PTL with ATO, and expected an improvement in the anti-HCC effect of ATO. Unexpectedly, we found that PTL significantly promotes the anti-HCC effect of ATO *in vivo* and *in vitro* by further inhibiting the proliferation and self-renewal ability of HCC, blocking cell cycle, enhancing the production of ROS, and inducing mitochondrial-dependent apoptosis. Furthermore, low dose of PTL (10 μM) used does not dramatically affects the human hepatocyte LO2 cells and HCC cell lines, instead, enhances the efficacy of ATO, indicating that PTL may be a more advantageous agent combined with ATO than other chemotherapeutics in treatment of HCC.

PTL is an inhibitor of NF-kB pathway and USP7 (33, 49), but ATO activates or inhibits NF-kB activity, depending on a cell type and the drug dose used (33, 50–52). Thus, we first focused on USP7-HUWE1-p53 axis to explore the mechanisms of combination therapy. Our results elucidated that single-agent ATO has no effect on the expression of USP7 and HUWE1, and the accumulation of p53 by ATO is evoked at transcript, but, PTL leads to the accumulation of p53 *via* weakening the interaction between USP7 and HUWE1, not at transcript (**Figure 5**, **S2A**). This conclusion revealed that combination of PTL and ATO can synergistically facilitate accumulation of p53 though two different pathways. Additionally, we found that PTL can not lead to the accumulation of p53 in combination with ATO in LO2 cells, and the detailed mechanism need to be further investigated (**Figure S2B**). On the other hand, the specific killing effect of PTL on cancer stem cells also contributes to the improvement of ATO in treatment of HCC. In this part, we just preliminarily explore the mechanism of combination therapy, and not further verify the conclusion in USP7-deficient cells or USP7+HUWE1 - deficient cells.

It is an effective strategy to eliminate the effect of antagonistic factors to antitumor drugs to improve clinical chemotherapy. Autophagy protects cancer cells from the application of chemotherapeutics, usually resulting in failure of therapy (38). Here, we first elucidated that combination of PTL and ATO can more obviously trigger autophagy in contrast with single agent, and weakening the autophagy further activates apoptosis induced by combined treatment. In addition, the conversion of LC3I to LC3II is not so obvious in MHCC 97H cell line, which was due to less sensitivity to drugs than other two cell lines (**Figures 1A-D**, **2A, B**). To verify the explanation, we increased the concentration of ATO in MHCC 97H cell line, and the results showed that combination of PTL and ATO observably increase the conversion of LC3I to LC3II (**Figure S1B**). CQ, a traditional antimalarial drug, is widely used as inhibitor of autophagy to treat various solid tumors in clinic. In animal experiment, application of CQ helps PTL and ATO further shrink the size of tumor, meaning that PTL and CQ combined with nontoxic dosage of ATO may benefit patients with HCC. Moreover, we confirmed that ATO combined with PTL has more significant ability of inhibiting cell growth than ATO combined with CQ (**Figure S2C**). PI3K/AKT/mTOR pathway, a critical pathway of treatment in HCC, is similarly involved in the activation of autophagy caused by combination of ATO/PTL, which provides possible directions for exploring the drugs aiming at this pathway, thereby enhancing the efficacy of ATO/PTL combination therapy.

In future work, we will further go on exploring the relationship between autophagy and apoptosis, and optimizing the combination of autophagy inhibitors and ATO/PTL to achieve the transformation from basic research to clinical application.

## Conclusions

All in all, our study demonstrated that PTL is the best candidate for optimizing the anti-tumor efficacy of ATO in application of hepatocellular carcinomas *in vitro* and *in vivo*. In addition, we also found USP7, a target protein of PTL, participates in the effect of PTL enhancing ATO-induced apoptosis to HCC cell lines *via* USP7-HUWE1-p53 axis. Meanwhile, our findings showed that the treatment of drug combination can more significantly enhance the activity of autophagy than that of each reagent, and the inhibition of autophagy using genetic or pharmacological methods can further improve the anti-tumor efficacy of a combination of ATO with PTL.

## Data availability statement

The original contributions presented in the study are included in the article/**Supplementary Material**. Further inquiries can be directed to the corresponding authors.

## Ethics statement

The animal study was reviewed and approved by Laboratory Animal Science and Technology Work Management Committee, School of Basic Medicine, Lanzhou University.

## Author contributions

Conceptualization, JY, XG, X-YY, and H-LW; methodology, JY and X-YY; soft-ware, J-XH and GC; validation, L-NW, LW, and X-YY; formal analysis, BX; investigation, JC; resources, JY; data curation, XG; writing—original draft preparation, JY, and X-YY; writing—review and editing, JY, XG, and H-LW; visualization, JY; supervision, JY, and H-LW; project administration, JY, XG, X-YY and H-LW; funding acquisition, JY, XG, and D-CW. All authors contributed to the article and approved the submitted version.

## Funding

This work was supported by the National Natural Science Foundation of China (NO.31701206); Natural Science Foundation of Gansu Province, China (No. 20JR5RA281 and NO. 21JR11RA089); Open Project of State Key Laboratory of Bioactive Substance and Function of Natural Medicines, Institute of Materia Medica, Chinese Academy of Medical Sciences and Peking Union Medical Colleges (No.GTZK 202006).

## Acknowledgments

We thank State Laboratory of Bioactive Substance and Function of Natural Medicines, Institution of Material Medica, Chinese Academy of Medical College for providing partial funding.

## Conflict of interest

D-CW was employed by LanZhou Ke Bao Biotechnology Co., Ltd.

The remaining authors declare that the research was conducted in the absence of any commercial or financial relationships that could be construed as a potential conflict of interest.

## Publisher’s note

All claims expressed in this article are solely those of the authors and do not necessarily represent those of their affiliated organizations, or those of the publisher, the editors and the reviewers. Any product that may be evaluated in this article, or claim that may be made by its manufacturer, is not guaranteed or endorsed by the publisher.

## References

[1] Antman KH. Introduction: The history of arsenic trioxide in cancer therapy. Oncologist (2001) 6(S2):1–2. doi: 10.1634/theoncologist.6-suppl_2-1 11331433

[2] Shen ZX Chen GQ Ni JH Li XS Xiong SM Qiu QY. Use of arsenic trioxide (As2O3) in the treatment of acute promyelocytic leukemia (APL): II. clinical efficacy and pharmacokinetics in relapsed patients. Blood (1997) 89(9):3354–60. 9129042

[3] Soignet SL Maslak P Wang ZG Jhanwar S Calleja E Dardashti LJ. Complete remission after treatment of acute promyelocytic leukemia with arsenic trioxide. N Engl J Med (1998) 339(19):1341–8. doi: 10.1056/NEJM199811053391901 9801394

[4] Soignet SL Frankel SR Douer D Tallman MS Kantarjian H Calleja E. United states multicenter study of arsenic trioxide in relapsed acute promyelocytic leukemia. J Clin Oncol (2001) 19(18):3852–60. doi: 10.1200/JCO.2001.19.18.3852 11559723

[5] Zhang S Ma C Pang H Zeng F Cheng L Fang B. Arsenic trioxide suppresses cell growth and migration via inhibition of miR-27a in breast cancer cells. Biochem Biophys Res Commun (2016) 469(1):55–61. doi: 10.1016/j.bbrc.2015.11.071 26592661

[6] Wang X Cao L Wu J Zhu G Zhu X Zhang X. Exploring the mechanisms of arsenic trioxide (Pishuang) in hepatocellular carcinoma based on network pharmacology. Evid Based Complement Alternat Med (2021) 2021:5773802. doi: 10.1155/2021/5773802 34880920 PMC8648446

[7] Li Z Zheng M Zhang H Yang X Fan L Fu F. Arsenic trioxide promotes tumor progression by inducing the formation of PGCCs and embryonic hemoglobin in colon cancer cells. Front Oncol (2021) 11:720814. doi: 10.3389/fonc.2021.720814 34676163 PMC8523995

[8] Noguera NI Catalano G Banella C Divona M Faraoni I Ottone T. Acute promyelocytic leukemia: Update on the mechanisms of leukemogenesis, resistance and on innovative treatment strategies. Cancers (Basel) (2019) 11(10). doi: 10.3390/cancers11101591 PMC6826966 31635329

[9] McCafferty-Grad J Bahlis NJ Krett N Aguilar TM Reis I Lee KP. Arsenic trioxide uses caspase-dependent and caspase-independent death pathways in myeloma cells. Mol Cancer Ther (2003) 2:1155–1164. 14617789

[10] Zhang Y Wu JH Han F Huang JM Shi SY Gu RD. Arsenic trioxide induced apoptosis in retinoblastoma cells by abnormal expression of microRNA-376a. Neoplasma (2013) 60(3):247–53. doi: 10.4149/neo_2013_033 23373993

[11] Zhang X Jia S Yang S Yang Y Yang T Yang Y. Arsenic trioxide induces G2/M arrest in hepatocellular carcinoma cells by increasing the tumor suppressor PTEN expression. J Cell Biochem (2012) 113(11):3528–35. doi: 10.1002/jcb.24230 22730174

[12] Pu YS Hour TC Chen J Huang CY Guan JY Lu SH. Arsenic trioxide as a novel anticancer agent against human transitional carcinoma--characterizing its apoptotic pathway. Anticancer Drugs (2002) 13(3):293–300. doi: 10.1097/00001813-200203000-00011 11984073

[13] Nasrollahzadeh A Bashash D Kabuli M Zandi Z Kashani B Zaghal A. Arsenic trioxide and BIBR1532 synergistically inhibit breast cancer cell proliferation through attenuation of NF-κB signaling pathway. Life Sci (2020) 257:118060. doi: 10.1016/j.lfs.2020.118060 32645343

[14] Zhou GB Zhao WL Wang ZY Chen SJ Chen Z. Retinoic acid and arsenic for treating acute promyelocytic leukemia. PloS Med (2005) 2(1):e12. doi: 10.1371/journal.pmed.0020012 15696202 PMC545204

[15] Zhao Z Zhang H Chi X Li H Yin Z Huang D. Silica nanovehicles endow arsenic trioxide with an ability to effectively treat cancer cells and solid tumors. J Mater Chem B (2014) 2(37):6313–23. doi: 10.1039/C4TB00874J 32262148

[16] Kim J Aftab BT Tang JY Kim D Lee AH Rezaee M. Itraconazole and arsenic trioxide inhibit hedgehog pathway activation and tumor growth associated with acquired resistance to smoothened antagonists. Cancer Cell (2013) 23(1):23–34. doi: 10.1016/j.ccr.2012.11.017 23291299 PMC3548977

[17] Beauchamp EM Ringer L Bulut G Sajwan KP Hall MD Lee YC. Arsenic trioxide inhibits human cancer cell growth and tumor development in mice by blocking Hedgehog/GLI pathway. J Clin Invest (2011) 121(1):148–60. doi: 10.1172/JCI42874 PMC3007144 21183792

[18] Zhang J Li C Zheng Y Lin Z Zhang Y Zhang Z. Inhibition of angiogenesis by arsenic trioxide via TSP-1-TGF-β1-CTGF-VEGF functional module in rheumatoid arthritis. Oncotarget (2017) 8(43):73529–46. doi: 10.18632/oncotarget.19867 PMC5650279 29088724

[19] Amigo-Jiménez I Bailón E Aguilera-Montilla N García-Marco JA García-Pardo A. Gene expression profile induced by arsenic trioxide in chronic lymphocytic leukemia cells reveals a central role for heme oxygenase-1 in apoptosis and regulation of matrix metalloproteinase-9. Oncotarget (2016) 7:83359–77. doi: 10.18632/oncotarget.13091 PMC5347775 27829220

[20] Wang HY Zhang B. Zhou JN Wang DX Xu YC Zeng Q. Arsenic trioxide inhibits liver cancer stem cells and metastasis by targeting SRF/MCM7 complex. Cell Death Dis (2019) 10(6):453. doi: 10.1038/s41419-019-1676-0 31186405 PMC6560089

[21] Zheng JC Chang KJ Jin YX Zhao XW Li B Yang MH. Arsenic trioxide inhibits the metastasis of small cell lung cancer by blocking calcineurin-nuclear factor of activated T cells (NFAT) signaling. Med Sci Monit (2019) 25:2228–37. doi: 10.12659/MSM.913091 PMC6446656 30913205

[22] Wang W Adachi M Zhang R Zhou J Zhu D. A novel combination therapy with arsenic trioxide and parthenolide against pancreatic cancer cells. Pancreas (2009) 38(4):e114–23. doi: 10.1097/MPA.0b013e3181a0b6f2 19342982

[23] Zhang YL Qiao SK Guo X.N Ren JH Zhang JN. Arsenic trioxide-induced cell apoptosis and cell cycle arrest are potentiated by 1,25-dihydroxyvitamin D3 in human leukemia K562 cells. Oncol Lett (2021) 22(1):509. doi: 10.3892/ol.2021.12770 34025784 PMC8130051

[24] Wei HL Yao XJ Li YN Wang P Zhao HS Bai DC. Arsenic trioxide inhibits p-glycoprotein expression in multidrug-resistant human leukemia K562/ADM cell line that overexpresses mdr-1 gene and enhances their chemotherapeutic sensitivity. Zhonghua Xue Ye Xue Za Zhi (2003) 24(1):28–31. 12679007

[25] Liu JX Zhou GB Chen SJ Chen Z. Arsenic compounds: revived ancient remedies in the fight against human malignancies. Curr Opin Chem Biol (2012) 16(1-2):92–8. doi: 10.1016/j.cbpa.2012.01.015 22342767

[26] Subbarayan PR Ardalan B. In the war against solid tumors arsenic trioxide needs partners. J Gastrointest Cancer (2014) 45(3):363–71. doi: 10.1007/s12029-014-9617-8 24825822

[27] Lin CC Hsu C Hsu CH Hsu WL Cheng AL Yang CH. Arsenic trioxide in patients with hepatocellular carcinoma: a phase II trial. Invest New Drugs (2007) 25(1):77–84. doi: 10.1007/s10637-006-9004-9 16937079

[28] Fang LJ Shao XT Wang S Lu GH Xu T Zhou JY. Sesquiterpene lactone parthenolide markedly enhances sensitivity of human A549 cells to low-dose oxaliplatin via inhibition of NF-kappaB activation and induction of apoptosis. Planta Med (2010) 76(3):258–64. doi: 10.1055/s-0029-1186083 19774508

[29] Schneider-Stock R Ghantous A Bajbouj K Saikali M Darwiche N. Epigenetic mechanisms of plant-derived anticancer drugs. Front Biosci (Landmark Ed) (2012) 17(1):129–73. doi: 10.2741/3919 22201736

[30] Guzman ML Rossi RM Karnischky L Li X Peterson DR Howard DS. The sesquiterpene lactone parthenolide induces apoptosis of human acute myelogenous leukemia stem and progenitor cells. Blood (2005) 105(11):4163–9. doi: 10.1182/blood-2004-10-4135 PMC1895029 15687234

[31] Kim K Doi A Wen B Ng K Zhao R Cahan P. Epigenetic memory in induced pluripotent stem cells. Nature (2010) 467(7313):285–90. doi: 10.1038/nature09342 PMC3150836 20644535

[32] Ghantous A Gali-Muhtasib H Vuorela H Saliba NA Darwiche N. What made sesquiterpene lactones reach cancer clinical trials? Drug Discov Today (2010) 15(15-16):668–78. doi: 10.1016/j.drudis.2010.06.002 20541036

[33] Li X Kong L Yang Q Duan A Ju X Cai B. Parthenolide inhibits ubiquitin-specific peptidase 7 (USP7), wnt signaling, and colorectal cancer cell growth. J Biol Chem (2020) 295(11):3576–89. doi: 10.1074/jbc.RA119.011396 PMC7076227 32029476

[34] Yi J Wang L Wang XY Sun J Yin XY Hou JX. Suppression of aberrant activation of NF-κB pathway in drug-resistant leukemia stem cells contributes to parthenolide-potentiated reversal of drug resistance in leukemia. J Cancer (2021) 12(18):5519–29. doi: 10.7150/jca.52641 PMC8364658 34405014

[35] Liu M Yang Y Liu D Cao Y Li Y. Parthenolide increases the sensitivity of gastric cancer cells to chemotherapy. J Tradit Chin Med (2020) 40(6):908–16. doi: 10.19852/j.cnki.jtcm.2020.06.002 33258341

[36] Li CJ Guo SF Shi TM. Culture supernatants of breast cancer cell line MDA-MB-231 treated with parthenolide inhibit the proliferation, migration, and lumen formation capacity of human umbilical vein endothelial cells. Chin Med J (Engl) (2012) 125(12):2195–9. 22884152

[37] Wright TJ McKee C Birch-Machin MA Ellis R Armstrong JL Lovat PE. Increasing the therapeutic efficacy of docetaxel for cutaneous squamous cell carcinoma through the combined inhibition of phosphatidylinositol 3-kinase/AKT signalling and autophagy. Clin Exp Dermatol (2013) 38(4):421–3. doi: 10.1111/ced.12138 23530461

[38] Rosenfeld MR Ye X Supko JG Desideri S Grossman SA Brem S. A phase I/II trial of hydroxychloroquine in conjunction with radiation therapy and concurrent and adjuvant temozolomide in patients with newly diagnosed glioblastoma multiforme. Autophagy (2014) 10(8):1359–68. doi: 10.4161/auto.28984 PMC4203513 24991840

[39] Su YC Davuluri GV Chen CH Shiau DC Chen CC Chen CL. Galectin-1-Induced autophagy facilitates cisplatin resistance of hepatocellular carcinoma. PloS One (2016) 11(2):e0148408. doi: 10.1371/journal.pone.0148408 26859293 PMC4747500

[40] Xu Y Yu H Qin H Kang J Yu C Zhong J. Inhibition of autophagy enhances cisplatin cytotoxicity through endoplasmic reticulum stress in human cervical cancer cells. Cancer Lett (2012) 314(2):232–43. doi: 10.1016/j.canlet.2011.09.034 22019047

[41] Stringer BW Day BW D'Souza RCJ Jamieson PR Ensbey KS Bruce Z. A reference collection of patient-derived cell line and xenograft models of proneural, classical and mesenchymal glioblastoma. Sci Rep (2019) 9(1):4902. doi: 10.1038/s41598-019-41277-z 30894629 PMC6427001

[42] Cong L Zhang F. Genome engineering using CRISPR-Cas9 system. Methods Mol Biol (2015) 1239:197–217. doi: 10.1007/978-1-4939-1862-1_10 25408407

[43] Cheng G Xie L. Parthenolide induces apoptosis and cell cycle arrest of human 5637 bladder cancer cells in vitro. Molecules (2011) 16(8):6758–68. doi: 10.3390/molecules16086758 PMC6264178 21829151

[44] Brown J Pagano M. Mechanism of p53 degradation. Biochim Biophys Acta (1997) 1332:O1–6. doi: 10.1016/S0304-419X(96)00048-0 9141469

[45] Kurokawa M Kim J Geradts J Matsuura K Liu L Ran X. A network of substrates of the E3 ubiquitin ligases MDM2 and HUWE1 control apoptosis independently of p53. Sci Signal (2013) 6(274):ra32. doi: 10.1126/scisignal.2003741 23652204 PMC3770270

[46] Khoronenkova SV Dianov GL. USP7S-dependent inactivation of mule regulates DNA damage signalling and repair. Nucleic Acids Res (2013) 41(3):1750–6. doi: 10.1093/nar/gks1359 PMC3561956 23275561

[47] Heras-Sandoval D Pérez-Rojas JM Hernández-Damián J Pedraza-Chaverri J. The role of PI3K/AKT/mTOR pathway in the modulation of autophagy and the clearance of protein aggregates in neurodegeneration. Cell Signal (2014) 26(12):2694–701. doi: 10.1016/j.cellsig.2014.08.019 25173700

[48] Whittaker S Marais R Zhu AX. The role of signaling pathways in the development and treatment of hepatocellular carcinoma. Oncogene (2010) 29(36):4989–5005. doi: 10.1038/onc.2010.236 20639898

[49] Bork PM Schmitz ML Kuhnt M Escher C Heinrich M. Sesquiterpene lactone containing Mexican Indian medicinal plants and pure sesquiterpene lactones as potent inhibitors of transcription factor NF-kappaB. FEBS Lett (1997) 402(1):85–90. doi: 10.1016/S0014-5793(96)01502-5 9013864

[50] Kerbauy DM Lesnikov V Abbasi N Seal S Scott B Deeg HJ. NF-kappaB and FLIP in arsenic trioxide (ATO)-induced apoptosis in myelodysplastic syndromes (MDSs). Blood (2005) 106(12):3917–25. doi: 10.1182/blood-2005-04-1424 PMC1895102 16105982

[51] Lemarie A Morzadec C Mérino D Micheau O Fardel O Vernhet L. Arsenic trioxide induces apoptosis of human monocytes during macrophagic differentiation through nuclear factor-kappaB-related survival pathway down-regulation. J Pharmacol Exp Ther (2006) 316(1):304–14. doi: 10.1124/jpet.105.092874 16174796

[52] Mathas S Lietz A Janz M Hinz M Jundt F Scheidereit C. Inhibition of NF-kappaB essentially contributes to arsenic-induced apoptosis. Blood (2003) 102(3):1028–34. doi: 10.1182/blood-2002-04-1154 12676792

