# Supplementary figures and images for: Parthenolide and arsenic trioxide co-trigger autophagy-accompanied apoptosis in hepatocellular carcinoma cells
Source: Front Oncol. 2022 Oct 24;12:988528. doi: 10.3389/fonc.2022.988528 (PMC9638029; doi:10.3389/fonc.2022.988528)

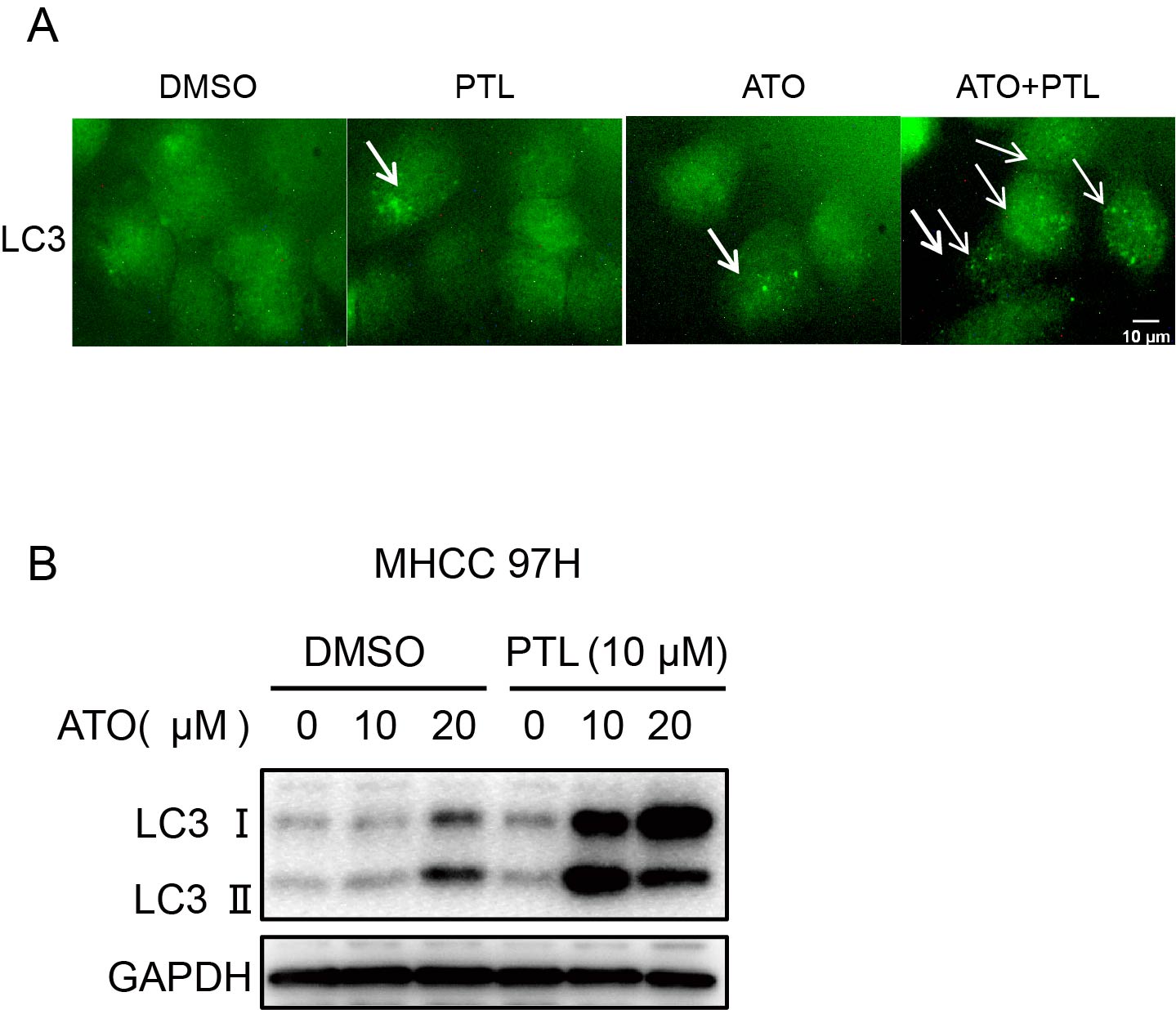

Supplement: Supplementary file 1 [file Image_1.jpg]

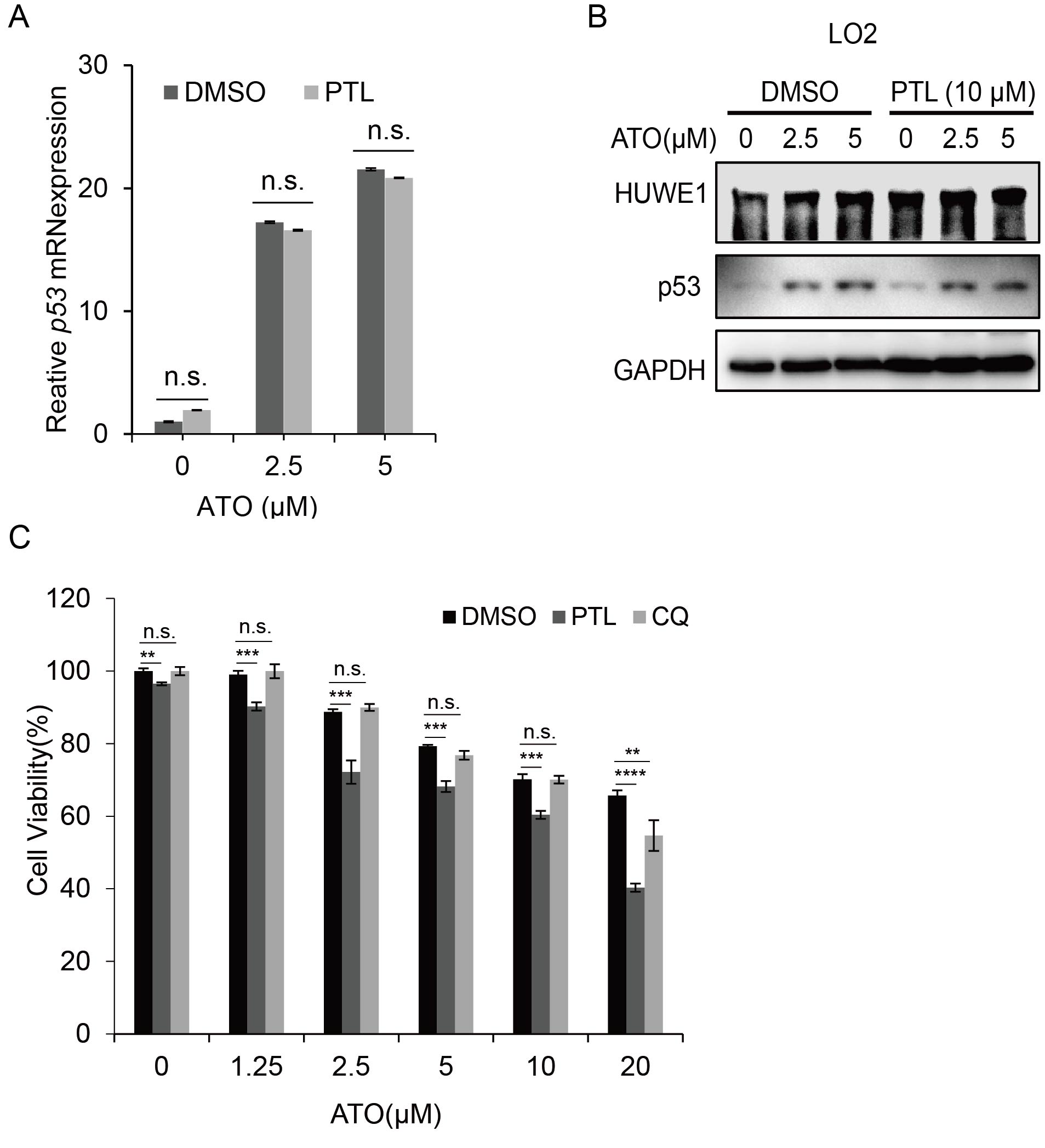

Supplement: Supplementary file 2 [file Image_2.jpg]
