# Supplementary material for: Parthenolide and arsenic trioxide co-trigger autophagy-accompanied apoptosis in hepatocellular carcinoma cells
Source: Front Oncol. 2022 Oct 24;12:988528. doi: 10.3389/fonc.2022.988528 (PMC9638029; doi:10.3389/fonc.2022.988528)
Supplement: Supplementary file 3 [file Presentation_1.pdf]

Supporting information

Materials and Methods

Immunostaining

To measure activity of autophagy by immunostaining, cells were seeded to a cover glass slide at the confluency of approximately 60%. After designated treatment, cells were fixed for 15 min at room temperature with 4% paraformaldehyde (PFA; Santa Cruz Biotechnology, sc-281,692), and then permeabilized with 0.25% TritonX-100 for 5 min at room temperature. Then, cells were blocked with 1% BSA (Sigma-Aldrich, A2153) for 1 h at room temperature. For immunostaining, cells were incubated with specific primary antibodies overnight at 4 °C. In the next day, the antibodies were removed and cells were rinsed with 0.1% Tween-20 in PBS (PBST) followed by incubation with anti-rabbit or anti-mouse Alexa-Fluor-488-conjugated secondary antibody for 1 h at room temperature. After that, cells were rinsed again with PBST three times and the cover glass slides were sealed with mounting medium containing DAPI (Thermo Fisher Scientific, P36980). The images were obtained with a confocal microscope (Olympus AX80, Tokyo, Japan).

RT－qPCR

HepG2 cells were seeded into 6-well for 24 h, and then were harvested and washed twice with PBS, thereafter, total RNA from cells was extracted using TRIZOL (Invitrogen, Thermo Fisher Scientific, Waltham, MA, USA). RT-qPCR was performed according to the manufacturer’s instructions (Yeasen, Shanghai, China). *p53* mRNA level was quantified by quantitative PCR (ROCGENE, Beijing, China) using the Archimed X5 real-time PCR detection system (ROCGENE, Beijing, China). The primers used were: *p53* (sense primer, 5-TGCGTGGAGTATTTGGATG-3′, and antisense primer, 5-TGGTACAGTCAGAGCCAACCTC-3′) and *GAPDH* (sense primer, 5′-ATGGCACCGTCAAGGCTGAG-3′, and antisense primer, 5′-GCAGTGATGGCATGGACTGT-3′).

Cell Counting Kit-8 (CCK-8) Assay

After treatment, the cells in 96 well were mixed with 10μL of CCK-8 solutions per well and incubated for further 2 h at 37°C. Optical density was measured at 450nm using a microplate reader (Powerwave X, Bio-Tek) to calculate inhibition rate for cell proliferation.

Figure Legend

Figure S1. PTL enhances ATO-triggered protective autophagy. (A) HepG2 cells were treated with ATO, PTL and ATO plus PTL for 24 h. The cells were subjected to immunofluorescence analysis after staining for LC3 antibody (Green). Scale bar: 10 μm. (B) MHCC97H cells were treated with ATO, PTL and ATO plus PTL for 24 h. Cells were harvested and subjected to western blotting analysis.

Figure S2. (A) HepG2 cells were treated with ATO, PTL and ATO plus PTL for 24 h. The level of p53 mRNA was detected by RT-qPCR, and were quantified; Columns, mean; bars, ± S.D. of 3 independent experiments (n.s. means *no significance*). (B) LO2 cells were treated with ATO, PTL and ATO plus PTL for 24 h. Cells were harvested and subjected to western blotting analysis. (C) LO2 cells were treated different concentrations of ATO plus PTL or ATO plus CQ for 24 h , and the cell viability was measured by CCK8 assay. Columns, mean; bars, ± S.D. of 3 independent experiments (**, $P < 0.01$; ***, $P < 0.001$; ****, $P < 0.0001$; n.s. means *no significance*).
